# A Narrative Review on Endomyocardial Fibrosis: Unraveling an Under-Recognized Tropical Heart Disease

**DOI:** 10.7759/cureus.96651

**Published:** 2025-11-12

**Authors:** Shushrusha Adhikari, Deeyar Ibrahim, Sri Lakshmi Kothakapa, Luis Pena, Tanvi Barsinge, Uzair Nisar Malik, Stephanie Nwokeji, Pranav Raj, Ayush Mittal, Ahmed Hassan Helmy Elsayed Fetih, Sara Razi

**Affiliations:** 1 Internal Medicine, Kathmandu Medical College, Kathmandu, NPL; 2 Internal Medicine, Wirral University Teaching Hospital, Birkenhead, GBR; 3 Emergency Medicine, Malla Reddy Institute of Medical Sciences, Hyderabad, IND; 4 Internal Medicine, Jersey City Medical Center, Jersey City, USA; 5 Internal Medicine, Seth Gordhandas Sunderdas Medical College and King Edward Memorial (KEM) Hospital, Mumbai, IND; 6 Internal Medicine, Northwick Park Hospital, Harrow, GBR; 7 Oncology, MD Anderson Cancer Center, Rush University Medical Center, Chicago, USA; 8 Internal Medicine, Government Medical College Patiala, Patiala, IND; 9 Internal Medicine, Maharaja Agrasen Medical College, Hisar, IND; 10 Internal Medicine, Faculty of Medicine Kasr Al-Ainy, Cairo University, Cairo, EGY; 11 Medicine and Surgery, Islamic Azad University, Tehran, IRN

**Keywords:** cardiac mri, echocardiography, endomyocardial fibrosis, multimodality imaging, neglected diseases, restrictive cardiomyopathy, schistosomiasis, thrombus, uganda

## Abstract

Endomyocardial fibrosis (EMF) is a neglected tropical cardiomyopathy, primarily affecting children and young adults in low-income equatorial regions. Characterized by fibrotic thickening of the ventricular endocardium, EMF leads to restrictive physiology, atrioventricular valve dysfunction, and progressive heart failure. Despite being recognized for over half a century, its etiology remains poorly understood, with proposed contributors including malnutrition, parasitic infections, hypereosinophilia, and genetic predisposition. Diagnosis is often delayed due to overlap with other causes of heart failure and limited access to advanced imaging. Echocardiography remains the primary diagnostic tool, especially in resource-limited settings, while cardiac MRI offers superior tissue characterization. Treatment is largely symptomatic, with diuretics, anticoagulants, and antiarrhythmics offering palliative benefit. Surgical intervention-particularly endocardial decortication and valve repair-remains the definitive treatment for advanced cases, though it carries high operative risk and is often inaccessible. Emerging approaches, such as artificial intelligence (AI)-assisted imaging and radiomics, hold promise for early detection. This narrative review synthesizes current knowledge on EMF’s epidemiology, pathophysiology, diagnosis, and management, underscoring the urgent need for improved awareness, research funding, and global health prioritization to mitigate its burden in vulnerable populations.

## Introduction and background

Endomyocardial fibrosis (EMF) is a form of restrictive cardiomyopathy that is often associated with fibrous deposits within the endomyocardium, particularly at the apices and inflow tracts of both the right ventricle (RV) and left ventricle (LV) [1]. The scarring produced by fibrous deposits leads to impaired diastolic filling and atrioventricular (AV) valve regurgitation [2], with more than half of reported EMF cases originating in sub-Saharan Africa, predominantly affecting underprivileged children and young adults [3].

In hospital practice, cases are often overlooked due to symptom overlap with other conditions that involve heart failure (HF), ascites, and peripheral edema. Given the disease's clinical characteristics, various diagnostic tests, including cardiac catheterizations and paracentesis, are usually performed. Nevertheless, the most reliable diagnostic method to confirm the diagnosis is a biopsy, typically revealing subendocardial fibrosis in conjunction with the presence of mural thrombi [4]. Although its etiology remains unclear, numerous theories concerning its causation have emerged, including links to environmental exposure, infectious agents, nutritional deficiencies, and autoimmune responses [1].

In endemic regions, EMF exacts a heavy clinical toll. EMF’s global burden might rival that of Chagas cardiomyopathy [1]. For example, up to 20% of chronic HF cases in certain Mozambican and Ugandan cohorts were attributed to EMF [3]. In Kampala, Uganda, EMF accounted for roughly one-fifth of patients referred for echocardiography (ECHO) [1], and in Nigeria, it has become one of the leading causes of heart disease (the fourth most common in one series) [5]. Nevertheless, progress over the years has introduced new techniques like ECHO and cardiac magnetic resonance (CMR), facilitating a more comprehensive examination for this condition [6].

The prognosis is grim. An autopsy series from Uganda reported an average survival of only ~2 years after symptom onset, and modern cohorts find 30%-50% mortality within two years in advanced cases [1]. Surgical endocardial resection and valve repair can substantially extend life in selected patients [3], but such operations carry high risk and are rarely available in resource-poor settings where EMF persists.

Once a diagnosis is established, significant health complications and the risk of death associated with arrhythmias and thrombotic events may follow [7]. The exact causes and progression are uncertain, and the natural course remains unclear. As a result, EMF is notably absent from major cardiology guidelines and global health agendas, reflecting how overlooked it has become [8]. This narrative review aims to comprehensively study the aspects of this disease, encompassing its etiology, diagnosis, and management, to improve physicians' comprehension and awareness of EMF. Relevant literature was identified through PubMed, Scopus, and Google Scholar using keywords such as “Endomyocardial Fibrosis” and “Restrictive Cardiomyopathy.” Articles published in English within the last 10 decades were reviewed and synthesized narratively.

## Review

Epidemiology and geographic distribution

EMF remains the most prevalent form of restrictive cardiomyopathy among children and young adults living within 15 degrees of the equator, with major endemic foci reported across East and West Africa, South Asia, and parts of South America [9,10]. The disease remains particularly concentrated in Uganda, Mozambique, Nigeria, and the coastal regions of Kerala, India, as well as in Bahia, Brazil, and Guangxi, China [11,12].

Although earlier reports identified these regions as EMF “hotspots,” recent hospital-based series from Uganda and India indicate a decline in new diagnoses, likely reflecting improvements in nutrition, sanitation, and reduced parasitic infections [13-15]. However, these findings are largely based on hospital registries rather than population-level surveillance; thus, true incidence trends remain uncertain. In contrast, a large community-based ECHO screening study in rural Mozambique found an overall prevalence of 19.8% (95% CI, 17.4-22.2), emphasizing that subclinical or asymptomatic disease may persist despite apparent declines in clinical cases [16].

Discrepancies between hospital-based and community-based prevalence estimates highlight potential bias-hospital data tend to capture more severe or late-stage cases, while community surveys detect early or asymptomatic forms. Environmental hypotheses such as magnesium deficiency and cerium exposure have been proposed but remain supported mainly by small observational and ecological studies with limited causal strength [17-19].

Familial clustering of EMF further supports a gene-environment interaction. The first genetic investigation by Beaton et al. demonstrated HLA-B58 and HLA-A02:02 associations in Mozambique and Uganda, respectively [20], yet no genome-wide or exome-based studies have since validated these findings, underscoring the need for broader genetic exploration.

Sex distribution also varies geographically-female predominance is noted in Uganda [10], while male predominance is reported in Mozambique and Nigeria [17,21,22]. These differences likely stem from nutritional, hormonal, occupational, and sociocultural factors rather than a uniform biological effect.

Despite advances in diagnosis and surgery, long-term prognosis remains poor in advanced disease, with historical data showing two-year mortality approaching 75% [23]. EMF continues to account for up to 20% of HF admissions in endemic African regions and represents the second leading cause of pediatric admission for acquired heart disease after rheumatic heart disease [12,24-26].

Etiology and pathophysiology

Although EMF has been recognized for over half a century [27], its precise pathogenesis remains incompletely understood. Current evidence supports a multifactorial model in which nutritional deficiencies, infections with associated eosinophilia, immune dysregulation, and genetic predisposition interact within adverse socioeconomic settings to promote progressive endocardial fibrosis [28,29].

Malnutrition and micronutrient imbalance appear consistently associated with EMF in endemic, low-income regions [8,29]. Diets deficient in magnesium and protein but rich in cerium and cassava-derived cyanogenic glycosides are hypothesized to trigger oxidative stress, endothelial injury, and fibroblast activation [8,18]. Experimental data demonstrate that magnesium deficiency promotes collagen deposition and pro-fibrotic cytokine release (TGF-β, IL-6), whereas cerium exposure induces myocardial oxidative injury and altered calcium signaling in animal models [18,27]. Nonetheless, human epidemiological confirmation remains limited.

Parasitic infections-particularly schistosomiasis and filariasis-are common cofactors in endemic zones [3,27]. Hypereosinophilia likely contributes to early myocardial necrosis through eosinophil granule proteins (major basic protein, eosinophil peroxidase), initiating an inflammatory-fibrotic cascade resembling Loeffler’s endocarditis [8,30]. However, eosinophil infiltration predominates in early disease, declining in chronic fibrotic stages, and parasitic load does not consistently differ between EMF and control subjects [29,30]. This suggests eosinophilia acts as a disease amplifier rather than a universal initiator.

Poverty-related malnutrition, recurrent infections, and delayed healthcare access perpetuate chronic immune activation and tissue remodeling [7,8]. Inflammatory cytokine profiling from limited contemporary cohorts shows elevated TGF-β1 and IL-10, consistent with a Th2-skewed, pro-fibrotic immune milieu [28]. Thus, socioeconomic deprivation magnifies biological susceptibility through nutritional and immunological pathways rather than acting independently.

Familial clustering and ethnic concentration suggest heritable susceptibility [29]. The only formal genetic study to date identified associations between EMF and HLA-B58 in Mozambique and HLA-A02:02 in Uganda [27], but no genome-wide or sequencing-based analyses have been published. Lack of replication and small sample sizes limit inference. Molecular studies implicate dysregulation of fibrosis-related signaling (TGF-β/SMAD and matrix metalloproteinases), but these remain exploratory.

Clarifying causality requires integrated molecular-epidemiologic studies combining environmental exposure data, eosinophil biomarkers, and genomic profiling in prospective community cohorts. Particular emphasis should be placed on dissecting the mechanistic link between nutritional deficiencies, oxidative stress, and endocardial fibroblast activation to differentiate association from pathogenesis (Table 1).

**Table 1:** EMF multifactorial etiology EMF: endomyocardial fibrosis

Etiology	Proposed mechanism
Genetic	Familial and ethnic clustering–possible HLA associations (HLA-B*58, HLA-A*02:02)
Infective/parasitic	Schistosomiasis, malaria, toxoplasmosis–associated hepatosplenic disease and ascites
Autoimmune	Mechanism proposed–minimal evidence
Immunological/eosinophilic	Hypereosinophilia as an independent risk factor. Similarities with Loeffler’s endocarditis
Nutritional	Malnutrition leading to immune dysregulation–diets low in magnesium (Mg) and protein. Additionally, diets high in cerium (Ce), vitamin D/E, or cyanogenic glycosides (cassava-rich diets)
Socioeconomic	Disease of the underprivileged–concentrated in low-income tropical regions with poor sanitation and limited healthcare access–decline in Kerala linked to improving standard of living

Pathogenesis

At the cellular level, EMF arises from a multifactorial interplay between inflammatory, immunologic, and fibrotic mechanisms. A key feature is fibroblast overactivation leading to excessive extracellular matrix deposition, primarily fibrillar collagen, which replaces normal myocardium and results in myocardial stiffness and electromechanical uncoupling. In vitro studies using cocultures of RT3 fibroblasts and SV40-transformed RL-14 cardiomyocytes [31-36] have provided partial mechanistic insights-demonstrating fibroblast migration, vimentin upregulation, inhibition of cardiomyocyte proliferation, and morphologic distortion of cardiomyocytes. However, these models serve only as mechanistic proxies rather than true EMF disease models. Their limitations include artificial cell lines, absence of eosinophils or endothelial cells, and lack of physiologic extracellular matrix composition, which restrict their translational validity to human disease.

In vitro studies using cocultures of RT3 fibroblasts and SV40-transformed RL-14 cardiomyocytes [31-36] have provided partial mechanistic insights-demonstrating fibroblast migration, vimentin upregulation, inhibition of cardiomyocyte proliferation, and morphologic distortion of cardiomyocytes. However, these models serve only as mechanistic proxies rather than true EMF disease models. Their limitations include artificial cell lines, absence of eosinophils or endothelial cells, and lack of physiologic extracellular matrix composition, which restricts their translational validity to human disease.

Fibroblast-cardiomyocyte interactions in vivo likely occur via paracrine signaling and mechanical feedback. Cytokine pathways such as TGF-β1, IL-4/IL-13, IL-5, IL-6, IL-10, and GM-CSF, along with chemokines like eotaxin (CCL11), have been implicated in promoting fibroblast activation, eosinophil recruitment, and matrix remodeling. Persistent activation of these pathways amplifies pro-fibrotic signaling (e.g., SMAD2/3 and YAP/TAZ), leading to chronic endocardial scarring [18,27-36].

Eosinophilic activity predominates during the early (acute) phase, causing direct cytotoxic injury through the release of major basic protein and eosinophil peroxidase. In later stages, eosinophilic infiltration subsides-likely due to clonal exhaustion, immune regulation, or treatment-while fibrotic repair becomes self-sustaining through fibroblast-matrix cross-talk and growth factor feedback loops.

The disease typically progresses through three overlapping phases: (1) acute phase: characterized by eosinophilic myocarditis, myocardial edema, and endocardial necrosis; may present with fever, myocarditic chest pain, and transient LV dysfunction; (2) transitional phase: marked by mural thrombus formation and early collagen deposition; imaging shows patchy late gadolinium enhancement (LGE) or apical thrombi on ECHO/CMR; and (3) chronic phase: defined by dense endocardial fibrosis, apical obliteration, and restrictive physiology; imaging reveals apical thickening, valve tethering, and occasionally dystrophic calcification [31-36].

Ventricular involvement varies: biventricular (BV) disease occurs in ~50% of cases, isolated LV disease in ~40%, and RV disease in ~10%. BV involvement is generally associated with worse diastolic dysfunction, AV valve regurgitation, and poorer long-term outcomes [31-36].

Pathologically, EMF is characterized by fibrotic thickening of the endocardium extending from the apices to the posterior mitral or tricuspid leaflets, typically sparing the outflow tracts. Common features include mural thrombi, dystrophic calcification, papillary muscle and chordal fibrosis, and subendocardial neovascularization. Microscopic findings vary by stage-acute lesions show eosinophilic infiltration and necrosis, while chronic lesions exhibit dense collagen, elastic fiber fragmentation, and endocardial thickening. Left- and right-sided lesions demonstrate similar histology but differ in distribution.

Imaging-pathology correlation is pivotal: ECHO and cardiac MRI reliably identify apical fibrosis, thrombi, and restrictive filling patterns corresponding to pathological severity. Integration of these findings may improve early diagnosis and staging of EMF.

Pathology

The gross and microscopic examination findings of EMF autopsy specimens have been summarized in Table 2 [7,8,18,29,30,37-42].

**Table 2:** Potential gross and microscopic examination findings of EMF in autopsy specimens EMF: endomyocardial fibrosis

Gross examination	Microscopic examination
Atrial dilatation	Endocardial thickening (due to excessive collagen deposition)
Atrioventricular (AV) valve annulus dilatation	Lymphocyte-predominant inflammatory infiltrates at the junction of the endocardium and myocardium
Decrease in the size of the ventricular cavity	Anomalous, lymphatic-rich vascular pattern in the endocardium
Apical thrombus (firm, red to tan, organized mass at the apex of the left ventricle)	Interstitial fibrosis and scar tissue formation near areas of subendocardial fibrosis

Clinical features and complications

The clinical manifestations of EMF vary according to disease duration, activity, and the pattern of ventricular involvement, categorized as isolated RV, isolated LV, or BV forms. Reported prevalence differs among cohorts, with BV involvement ranging from 45% to 60%, isolated RV from 25% to 35%, and isolated LV from 10% to 20%, reflecting regional variation and case-mix differences rather than uniform global estimates [43-46].

Typical pathognomonic features include apical endocardial thickening and obliteration, AV valve tethering (resulting in mitral or tricuspid regurgitation), and restrictive diastolic filling on imaging or hemodynamic assessment. In contrast, symptoms such as fatigue, dyspnea, cough, chest pain, or peripheral edema are non-specific and may occur in various cardiomyopathies [43-45].

RV EMF often presents with a loud tricuspid regurgitant murmur, prominent systolic jugular pulsation, and hepatosplenomegaly. LV EMF typically manifests as exertional dyspnea, orthopnea, paroxysmal nocturnal dyspnea, and an apical pansystolic murmur of mitral regurgitation [46,47]. BV disease combines these findings and commonly produces disproportionate ascites with minimal peripheral edema, a distinctive feature of EMF. The exudative nature of ascitic fluid-rich in protein and inflammatory cells-suggests a component of peritoneal inflammation superimposed on venous and lymphatic congestion, although histologic or imaging confirmation of peritoneal involvement remains inconsistent and requires further investigation [48,49].

The association of eosinophilia with EMF, historically emphasized in studies from the 1970s to 1980s, remains biologically plausible but poorly defined in modern cohorts. Limited contemporary data report variable eosinophil elevations (30%-50%) without consistent correlation to disease activity, underscoring the need for updated, prospective evaluation [49,50].

In chronic stages, low cardiac output and hepatic congestion contribute to cachexia, hypoalbuminemia, parotid enlargement, ascites, and clinical feminization. Early reports noted low testosterone levels and testicular atrophy in men, likely secondary to hepatic dysfunction [51]; however, these findings have not been validated in recent series. Similarly, skeletal muscle fibrosis described in small biopsies remains anecdotal, and larger studies are warranted to confirm systemic fibrosing involvement (Table 3) [49].

**Table 3:** Clinical features of endomyocardial fibrosis (EMF) JVP: jugular venous pressure

Form	Key characteristics
Right ventricular EMF	Features of tricuspid regurgitation (≈90%); dominant systolic jugular vein pulsation, third heart sound; systemic venous hypertension, raised JVP, hepatosplenomegaly; ascites without pedal edema, exophthalmos; non-specific GI symptoms; central cyanosis
Left ventricular EMF	Features of mitral regurgitation (apical pan-systolic murmur, 3rd heart sound); exertional dyspnea, orthopnea, paroxysmal nocturnal dyspnea; signs of left-sided heart failure
Biventricular EMF	Combination of right and left ventricular features; striking ascites with little or no peripheral edema
General/chronic features	Growth retardation, cachexia, hypoalbuminemia, generalized edema; parotid swelling, cyanosis, clubbing, testicular atrophy, feminization; possible systemic fibrosis and eosinophilia association

Stage Correlation

The acute/active phase is characterized by eosinophilic myocarditis, fever, and transient HF. The transitional phase is marked by mural thrombus, early fibrosis, and restrictive physiology. The chronic phase is defined by dense fibrosis, valvular tethering, arrhythmia, cachexia, and multiorgan sequelae.

Complications include progressive HF (primarily diastolic), thromboembolism, pericardial effusion, angina, and arrhythmias [43-47]. Reported event rates suggest thromboembolism in up to 15%-20%, atrial fibrillation in 30%-40%, and sudden cardiac death in 5%-10% of advanced cases, although these estimates are derived from limited regional data. Larger, standardized cohort studies are needed to quantify these risks and refine stage-specific prognostic models.

Diagnostic modalities

Echocardiography

ECHO remains the gold standard for diagnosing EMF due to its non-invasive nature, widespread availability, and high concordance with surgical and autopsy findings [52,53]. It plays a critical role in both initial evaluation and long-term monitoring, especially in resource-limited endemic areas where advanced imaging modalities may be inaccessible. Its portability and minimal requirement for consumables make it ideal for use in rural and underserved settings [54]. Moreover, ECHO showed high diagnostic reliability for severe EMF, with perfect agreement between preoperative imaging findings and surgical observations. Notably, ECHO severity classifications correlated strongly with histopathological findings, as all cases identified as severe on ECHO demonstrated extensive endocardial fibrosis upon tissue examination [53]. A systematic review published in 2024 by Sozzi et al. demonstrated ECHO as the most commonly employed imaging technique (51.9%) [55].

Transthoracic ECHO

Transthoracic ECHO (TTE) reveals hallmark features of EMF, including apical obliteration due to dense fibrotic tissue, AV valve regurgitation caused by leaflet tethering or restriction, and atrial enlargement from diastolic dysfunction and volume overload [56,57]. Additional signs may include mural thrombus, pulmonary hypertension, and a restrictive filling pattern. The "mushroom sign," indicating apical distortion from fibrosis, is another characteristic feature and remains primarily a descriptive feature of apical distortion and has not been formally validated as a diagnostic criterion [56]. Recent advancements propose ECHO-based scoring systems to assess disease severity and phenotype based on the extent and location of fibrosis. These systems aim to standardize diagnosis across endemic regions and facilitate early detection.

ECHO demonstrates high sensitivity and specificity for advanced EMF [52,53], but its ability to detect early or subclinical cases before significant fibrosis develops is limited. Tissue Doppler imaging, known for identifying subclinical myocardial changes, may further enhance diagnostic accuracy when paired with biomarkers such as N-terminal pro-B-type natriuretic peptide (NT-proBNP) [53,57]. These large-scale studies are still limited, so these approaches largely remain exploratory but may improve early detection in the future. In quantitative terms, Berensztein et al. reported a sensitivity of 94% and specificity of 86% for ECHO in surgically confirmed EMF, while Mocumbi et al. observed 83% concordance with histopathology, demonstrating its reliability [52,53].

Mocumbi et al. developed major and minor criteria along with severity scores for the diagnosis and assessment of EMF. The presence of two major criteria or one major criterion associated with two minor criteria confirmed the diagnosis [56]. Furthermore, disease severity was stratified using a 35-point scale as mild (score ≤ 8), moderate (9-15), and severe (≥16), and its phenotypical classification was categorized into three subtypes based on the distribution of fibrotic lesions as BV (symmetric involvement), LV predominant, or RV predominant [56]. The study was first developed in rural Mozambique with N = 1,063 patients screened. While this system made diagnosis more consistent and easier to compare across patients, the study did not include a full statistical validation against a gold standard test, but supported standardized case classification and prevalence reporting [17].

Later, a smaller surgical study from the same region, Mozambican surgical series (N = 29), demonstrated high agreement between preoperative ECHO and intraoperative/histologic findings. The results showed excellent agreement in 24 out of 29 cases; the ECHO diagnosis matched what was found in the heart tissue [53]. This confirmed that the scoring system works well in clear, advanced cases, although the study was small and focused mainly on children, limiting generalizability and providing no inter-rater reliability statistics.

Outside Mozambique, the Mocumbi criteria have been used informally in other EMF-affected areas, including Kerala (India) [16] and Uganda [58]. However, each region has adapted the system differently. Some Indian studies combine Mocumbi’s and Shaper’s criteria, while Ugandan teams often describe EMF based on typical ECHO features rather than a formal score [12,16]. Because of these heterogeneous adoptions, there is no single standardized or fully validated version of the scoring system used worldwide.

Recent reviews consistently highlight the need for multicenter validation of ECHO criteria across diverse endemic populations. Such efforts are essential to standardize case detection, minimize operator-dependent variability, and account for regional differences in EMF phenotype, including variations in ventricular involvement and a declining disease burden in certain areas [12].

To ensure global applicability, future validation studies should adopt multicenter, prospective designs that include populations from sub-Saharan Africa, South Asia, and South America. Both community-based and hospital cohorts should be represented to capture the full clinical spectrum of EMF.

**Figure 1:**
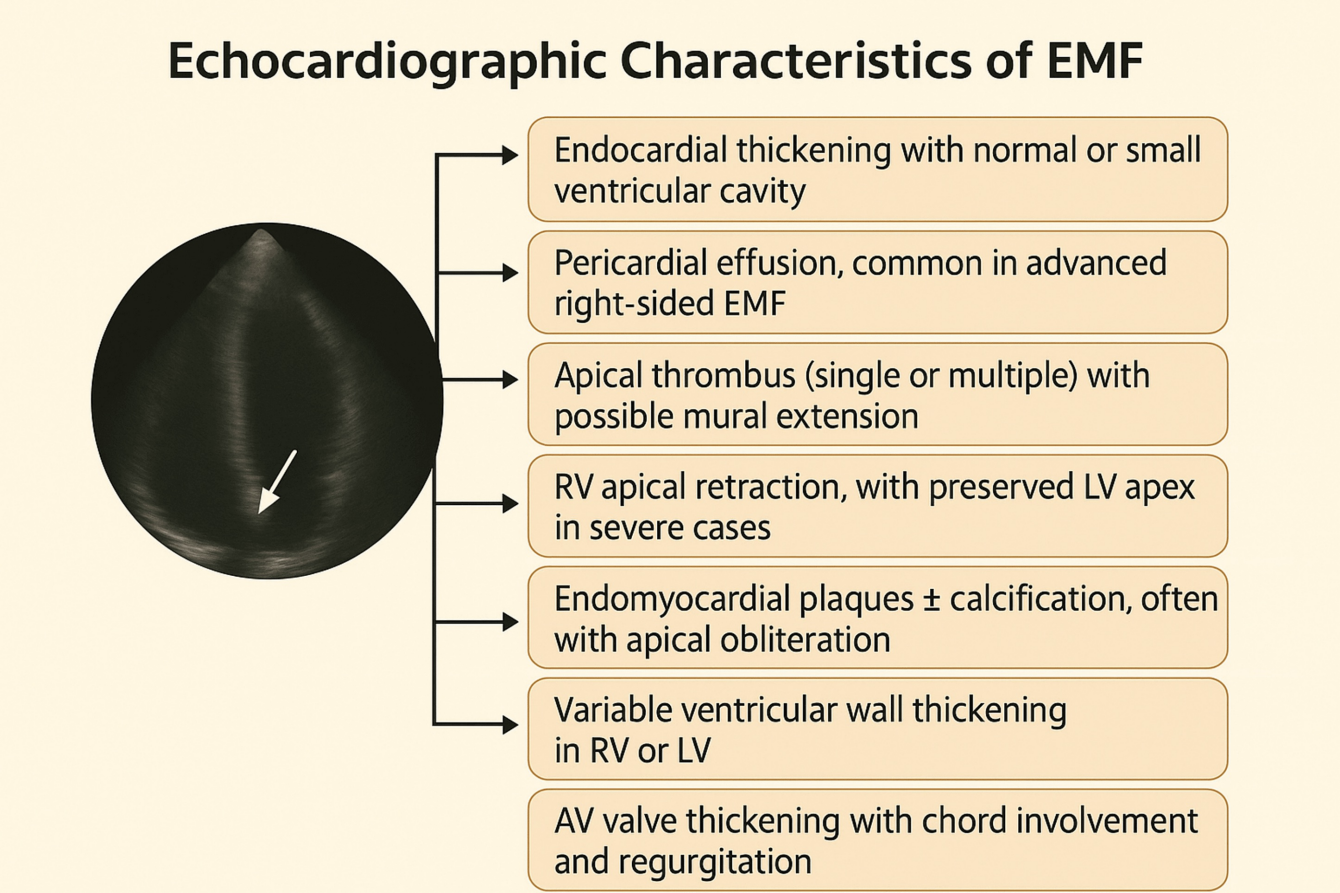
Echocardiographic characteristics of EMF EMF: endomyocardial fibrosis; LV: left ventricular; RV: right ventricular; AV: atrioventricular Credits: Sri Lakshmi Kothakapa, created with biorender.com

However, ECHO does have some limitations. It remains primarily a qualitative tool and lacks the ability to directly visualize or quantify myocardial fibrosis, a key pathological hallmark of EMF. Reproducibility can be affected by operator dependence and image quality, particularly in resource-limited settings [52,56,57]. Despite these limitations, it remains the most practical tool for longitudinal follow-up, enabling monitoring of disease progression and treatment response. Emerging techniques such as artificial intelligence (AI)-assisted ECHO analysis show promise in improving diagnostic accuracy and reproducibility, though they remain research tools (Figure 1) [59].

CMR Imaging

CMR imaging has emerged as a highly advanced and non-invasive imaging modality for evaluating cardiac structure, function, and tissue composition, offering superior resolution and diagnostic accuracy compared to traditional ECHO [60]. One of its most powerful features is its ability to provide detailed tissue characterization through techniques such as LGE, which enables visualization of myocardial fibrosis, inflammation, and thrombus formation [61].

In the context of EMF, LGE-CMR plays a pivotal diagnostic and prognostic role. A hallmark imaging feature is the continuous subendocardial enhancement extending from the subvalvular regions to the ventricular apices, reflecting fibrotic tissue accumulation. This pattern often includes a characteristic "double V" or "three-layered" sign, comprising normal myocardium, an intermediate zone of fibrotic endocardium, and an inner non-perfused apical thrombus, which correlates strongly with histopathological findings and aids in differentiating EMF from conditions such as rheumatic heart disease, Ebstein anomaly, hypertrophic cardiomyopathy, and tuberculous pericarditis [62,63]. This sign is highly indicative of EMF, but while it shows strong histopathological correlation, its sensitivity and specificity in differentiating EMF from other restrictive cardiomyopathies require further validation. CMR demonstrates superior diagnostic performance compared to ECHO and provides enhanced tissue characterization and high spatial resolution [53,62,63], although exact comparative quantitative metrics remain under investigation.

LGE-CMR capitalizes on differences in contrast kinetics between healthy and diseased myocardium. Gadolinium accumulates in fibrotic and necrotic tissue due to their expanded extracellular volumes, creating hyperenhancement on T1-weighted images [61]. An inversion recovery sequence nulls the signal from normal myocardium, enhancing lesion visibility. Limitations of LGE quantification in EMF include technical variability, suboptimal reproducibility, and patchy fibrosis distribution, which may lead to sampling errors [64,65]. Standardization of imaging protocols is crucial to mitigate these issues. LGE-CMR offers high spatial resolution for quantifying fibrotic burden, infarct size, and myocardial involvement, aiding diagnosis, risk stratification, and therapeutic monitoring [64]. The prognostic value of fibrosis burden has been validated. Salemi et al. [62] found that patients with extensive LGE-defined fibrosis experienced poorer functional recovery and higher mortality after surgery, indicating that fibrosis extent strongly correlates with adverse outcomes.

The volume of fibrosis indexed by LGE has been shown to independently predict mortality in EMF patients [62,63]. Additionally, CMR can identify myocardial edema and intracardiac thrombi, particularly in the right atrium, providing a comprehensive assessment when combined with 3D ECHO [65,66]. Despite its diagnostic superiority, feasibility and cost remain major constraints in endemic regions. León et al. [65] and Tai et al. [66] reported that while CMR allows earlier detection-even in pediatric and subclinical cases, its use is largely confined to tertiary centers due to equipment costs and limited access, challenging its widespread adoption in resource-limited settings.

Myocardial Contrast ECHO

Myocardial contrast ECHO (MCE) serves as a useful adjunct when conventional ECHO is limited by poor acoustic windows or coexistent thrombus and fibrosis. Clinical indications include detection of apical obliteration, intracavity thrombus, and subendocardial fibrosis that may be under-detected on routine ECHO [67]. MCE perfusion defects correlate with fibrotic areas seen on CMR, integrating MCE into diagnostic algorithms as a complementary tool rather than a primary modality. In a study by Wu et al., contrast-enhanced ECHO revealed clearer images of apical obliteration, intracavitary thrombus, and subendocardial fibrosis that were under-detected on routine imaging [67]. Perfusion defects noted on MCE corresponded well with regions of fibrosis and thrombus identified on CMR, highlighting its complementary role [67].

Endomyocardial Biopsy

While endomyocardial biopsy (EMB) provides histological confirmation, its use is limited in EMF due to procedural risks and the patchy distribution of fibrosis. EMB demonstrates high false-negative rates unless guided by imaging [68], and the current consensus recommends reserving EMB for diagnostically challenging cases or when differentiating from other cardiomyopathies.

Emerging Role of Radiomics and AI

Radiomics and AI are emerging tools in cardiac imaging, with potential for early EMF detection. By analyzing texture and shape features from ECHO and CMR, AI algorithms may detect subtle fibrotic changes. Current evidence is largely pilot-scale, with limited validation in clinical settings [59]. Barriers include software availability, integration into low-resource settings, and a lack of large-scale training datasets.

Management strategies

The management of EMF remains largely supportive and is guided by clinical severity and the availability of specialized care [69]. Diuretic therapy represents the cornerstone of symptom control, aiming to reduce venous congestion and relieve right-sided HF. Loop diuretics such as furosemide or torasemide are preferred, while thiazide-type agents or spironolactone are frequently added in resistant cases to augment natriuresis and counter secondary hyperaldosteronism [69-71]. Although the use of diuretics provides significant symptomatic improvement, their effect on disease progression is unproven. Dosing strategies are individualized and titrated according to clinical response, blood pressure, and renal function, as no standardized protocols exist due to wide variability in disease presentation [69-71].

Arrhythmias are common in EMF as a consequence of atrial dilation, myocardial fibrosis, and chronic pressure overload. Rate control with β-blockers or digoxin is often preferred, while rhythm-control strategies are limited by the high recurrence rate and potential proarrhythmic risk [70,72]. Pacemaker implantation is indicated for significant conduction abnormalities or symptomatic bradyarrhythmias, particularly following endocardiectomy. Implantable cardioverter-defibrillator (ICD) therapy has been rarely reported, and outcome data from endemic regions are lacking [72].

Anticoagulation plays a critical role in preventing systemic embolization in patients with atrial fibrillation or mural thrombus. Warfarin remains the primary anticoagulant, but its use in endemic regions is hindered by challenges in regular international normalized ratio (INR) monitoring, dietary variations, and cost [73]. Although direct oral anticoagulants (DOACs) offer potential advantages in safety and convenience, their clinical utility in EMF is limited by affordability, accessibility, and the absence of robust outcome data [73].

Surgical intervention remains the definitive therapy for patients with advanced disease (New York Heart Association (NYHA) class III-IV) who remain symptomatic despite optimal medical management and who demonstrate preserved ventricular geometry and satisfactory nutritional status [18,27,74]. Endocardiectomy with or without AV valve repair or replacement continues to provide the most substantial symptomatic and hemodynamic improvement when performed in experienced centers [74,75]. Survival outcomes vary significantly with surgical expertise and perioperative management, with reported five- and 10-year survival exceeding 60% in high-volume centers and markedly lower outcomes in early or low-experience series [74-78]. Beyond AV block and recurrence, perioperative complications include excessive bleeding, RV dysfunction, and low-cardiac-output syndrome, particularly among patients with advanced preoperative hemodynamic compromise [74,75].

In resource-limited settings, multidisciplinary coordination among cardiologists, cardiac surgeons, imaging specialists, and nutritionists is essential to optimize outcomes and improve long-term survival. For patients deemed unsuitable for surgery, comprehensive palliative care-including optimized diuretic therapy, rate control, anticoagulation, and nutritional rehabilitation-remains fundamental to preserving functional status and quality of life [18,27,74].

Prognosis

Prognosis is highly variable and strongly influenced by the stage at diagnosis, extent of ventricular involvement, and availability of surgical expertise. Historically, untreated EMF carried a grave outlook, with two-year survival rates as low as 5% [79]. In contrast, outcomes in contemporary surgical series have improved markedly, with experienced centers reporting five- to 10-year survival exceeding 60% following endocardial decortication and valve repair or replacement [74,75,79]. These discrepancies reflect both temporal advances in management and regional disparities in healthcare access and surgical capacity [18,27].

Several predictors of poor outcome have been identified. Advanced diastolic dysfunction, severe atrial enlargement, BV disease, and extensive subendocardial fibrosis or thrombus on ECHO or CMR imaging correlate with adverse prognosis [52,62-65]. Elevated HF biomarkers such as NT-proBNP, together with pulmonary hypertension or hepatic congestion, further signify disease progression and limited reversibility [53,57,79]. The observed association between EMF and aortic valve calcification or stenosis may exacerbate hemodynamic compromise and affect postoperative recovery, although its prognostic impact-particularly in patients undergoing transcatheter aortic valve replacement-remains incompletely characterized [78].

Recurrence of fibrosis after surgery occurs in approximately 6%-18.8% of patients, often within a few years of intervention [75,79]. Accordingly, structured long-term surveillance using periodic ECHO or CMR is essential to detect fibrotic relapse and valve dysfunction early. In endemic, resource-limited regions, prognosis remains considerably poorer owing to diagnostic delays, restricted surgical availability, and limited long-term care infrastructure [18,27]. Multidisciplinary management plays a crucial role in optimizing outcomes and improving the quality of life for patients who are inoperable or present with advanced disease.

Recent advances and research gaps

EMF, historically prevalent in Africa, Asia, and South America, has seen a regional decline, potentially due to improved healthcare and communicable disease control. At the Uganda Heart Institute, newly diagnosed EMF cases fell sharply over 14 years, indicating a sustained decrease in hospital-detected incidence [58]. In another study conducted in rural southern Mozambique, a high burden of silent disease was still observed, with about one in five individuals meeting EMF criteria, and most cases were asymptomatic at detection [17]. Environmental factors like schistosomiasis remain central to EMF pathogenesis, and integrated control programs, such as those in China, highlight the potential of sanitation and health education to reduce incidence [18,80]. Despite its impact, accurate prevalence data are scarce due to limited community-based research. Africa continues to carry a disproportionate burden of cardiovascular disease, including EMF, underscoring the need for targeted interventions aligned with global health goals [8]. Advances like point-of-care diagnostics, natriuretic peptides (BNP/NT-proBNP), and high-sensitivity cardiac troponin and imaging techniques offer promise for better research and management through international collaborations; however, these biomarkers are not specific for EMF and should be used for triage and monitoring rather than diagnosis [81]. In highly endemic regions like southern Mozambique, ECHO screening has shown potential for early detection, even in asymptomatic individuals [17]. AI-assisted ECHO interpretation can highly boost screening accuracy in related endemic diseases, such as rheumatic heart disease, but no EMF-specific approved AI model exists yet [82]. New studies should include practical execution of trials of handheld/tele-echo pathways (optionally AI-assisted) powered for clinical outcomes and incorporate economic evaluations tailored to local health systems [83]. However, challenges such as poor healthcare access, low awareness, and inadequate diagnostic tools hinder early diagnosis. Training local healthcare workers and deploying portable ECHO devices may enhance early detection and improve outcomes in low-resource settings [9].

## Conclusions

EMF is a neglected tropical cardiomyopathy that predominantly affects young people, especially women and children in low-resource equatorial regions. Despite decades of recognition, it remains a major cause of morbidity and early-onset HF due to limited awareness, diagnostic tools, and treatment access. ECHO continues to be the cornerstone of diagnosis in endemic areas, providing essential diagnostic accuracy and guidance for symptom management, while cardiac MRI offers superior tissue characterization that enhances risk stratification and optimizes timing of intervention. Although current medical management, including diuretics and HF therapy, mainly provides symptomatic relief without proven survival benefit, surgical approaches such as endocardial resection and valve repair have demonstrated improved survival and functional outcomes in selected patients, albeit with substantial procedural risk. Combining imaging modalities further refines diagnosis and guides therapy. Also, recognizing EMF as a public health priority is crucial, paralleling other neglected tropical cardiomyopathies. Strengthening local capacity through training in ECHO and CMR, deploying portable imaging in rural clinics, and implementing community screening can facilitate early detection. Future research should prioritize prognostic biomarkers, AI-assisted imaging, and standardized diagnostic criteria to ensure consistent global reporting and better outcomes.
